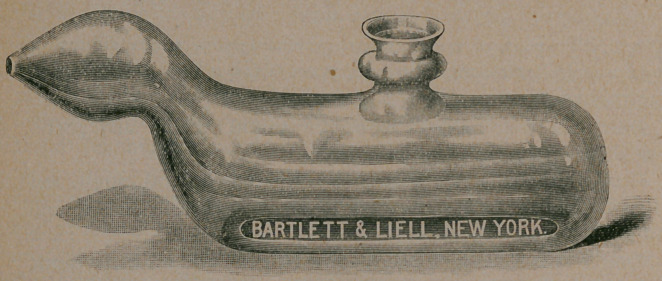# Rhinological Don’ts

**Published:** 1895-05

**Authors:** Edward J. Bermingham

**Affiliations:** Surgeon-in-Chief to the New York Throat and Nose Hospital; New York


For the Texas Medical Journal.

### RHINOLOGICAL DON'TS.

### What Not to Do in Nasal Affections.

BY EDWARD J. BERMINGHAM, A. M., M. D.,
Surgeon-in-Chief to the New York Throat and Nose Hospital.

Read before the Texas State Medical Association, April 23, 1895, and contributed by author to Texas Medical Journal.

DON'T speak of nasal catarrh as a disease. It is a symptom of irritation of the mucous membrane lining the nasal cavities, and has various causes.

DON'T make a diagnosis without a careful anterior and posterior rhinoscopic examination.

DON'T forget that the nose is meant to breathe through, and that complete or partial obstruction means mouth breathing and all its dangers.

DON'T fail to examine the nasal cavities in all cases of asthma, hay fever, deafness, and chronic cough.

DON'T use a palate hook in making a posterior rhinoscopic examination or post-nasal application. If the patient be trained to breathe through the nose, the soft palate will hang perpendicularly and the examination will be easy.

DON'T forget to cleanse the nasal cavities before making an examination or medicinal application. Medicated sprays or insufflations into the cavities lined with inspissated mucus are applied to the mucus and not to the membranes lining the cavities.

DON'T use salt water for cleansing, but an alkaline, non-irritating, antiseptic, and deodorizing solution of the proper specific gravity to promote osmosis. Bermingham's solution * meets the indications.

DON'T use a Thudicum douche or a syringe; nor any apparatus where the *force* of the stream is under the control of the patient. The Bermingham douche† is simple and has no objectionable features.

DON'T forget that all diseased conditions of the nasal mucous

---

| | |
|---|---|
| * Bicarbonate of soda | 2½ drachms |
| Biborate of soda | 2½ drachms |
| Salicylate of soda | 2½ drachms |
| Thymol | 2½ grains |
| Menthol | 2½ grains |
| Glycerine | 2½ ounces |
| Alcohol | 5 drachms |
| Distilled water, sufficient to make 8 ounces. | |
| Oleum pini Sylvestries | 5 drops. |

M. A half teaspoonful of this mixture should be added to four teaspoonfuls of tepid water, just before using in the nose.

† Dr. Bermingham's nasal douche is here represented exact size. It has a capacity of about seven drachms, generally sufficient for a thorough cleansing. It is to be used in the following manner: Fill the douche with the cleansing solution properly diluted, and at a temperature of about 100° F., close the funnel with the index finger, insert the nozzle into the nostril so that it closes the latter completely, throw the head slightly backward, raise the finger closing the funnel, and allow the solution to enter the nostril and flow through it to the naso-pharynx, around the posterior margin of the septum, until it emerges from the other nostril. This nostril should then be closed with the finger, so as to keep the nose filled with the solution, and the parts bathed in it for two or three minutes. The process should be repeated on the opposite side. Unless the patient breathes through the mouth quietly and naturally all the time he is using the douche, the solution will run down the throat. He will, however, become expert in using it in a day or two. After its use the nose and naso-pharynx should not be cleared for three minutes, when the solution will have drained away.

membrane will sooner or later produce middle ear disease; and that they may produce asthma and other reflex affections.

DON'T permit the patient to use cocaine under any circumstances.

DON'T use cocaine except for diagnostic or operative purposes.

DON'T forget that a five per cent. solution of antipyrine will contract the blood vessels, that its action is prolonged far beyond that of cocaine, and that the patient will never contract the cocaine habit by using it.

DON'T use cocaine in acute rhinitis. An antiseptic cleansing solution, followed by a spray of five per cent. solution of antipyrine, and small doses of quinine and belladonna internally, is the treatment indicated.

DON'T use irritating applications to the nasal mucous membrane in hypertrophic rhinitis. Cleansing is of the first importance in the treatment of this condition.

DON'T forget that a saturated solution of iodoform and tannin in ether is the best application to make to a diseased nasal mucous membrane. It should be made by means of the spray, both anteriorly and posteriorly to the pharyngeal vault.

DON'T abandon the iodoform treatment if you do not possess a spray apparatus. Use the compound stearate of zinc with iodoform in an insufflator.

DON'T discard the iodoform treatment in hypertrophic rhinitis unless the patient strenuously objects. Then try a five per cent. solution of antipyrine, or a one per cent. solution of menthol in albolene.

DON'T fail to see and treat all hypertrophic cases three times weekly, and have the patient cleanse thoroughly at home at least twice a day.

DON'T trust the patient, but ascertain, by examination at each visit, that he cleanses the nasal cavities thoroughly and properly.

DON'T forget that cleanliness is the *sine qua non* in the treatment of atrophic rhinitis. If it be neglected, all other treatment will fail.

DON'T fail to operate and restore the calibre of the nasal passages in all cases of stenosis causing total or partial obstruction of nasal respiration.

DON'T cut or cauterize unless stenosis exists to a degree to obstruct respiration.

DON'T hope to relieve catarrhal symptoms if stenosis exists, unless you correct the stenosis.

DON'T fail to distinguish between hypertrophy of the turbinated bone and hypertrophy of the tissues covering the bone. The differentiation can be made in a few minutes by the application of cocaine and pressure with a probe.

DON'T treat chronic hypertrophy of the tissues covering the turbinated bones with astringents. Destroy a portion of the tissue with the galvano-cautery if the hypertrophy is anterior. Remove it with the Jarvis' snare if it is posterior.

DON'T treat hypertrophy of the turbinated bones with the cautery. Remove a portion of the entire length of the bone with a saw if the inferior is affected; with the wire écraseur if the middle is affected.

DON'T use complicated instruments where simple ones will answer.

DON'T use a saw to remove small spurs and crests on the cartilaginous septum. The Chappell annular knife will answer better.

DON'T use the electric drill or trephine to remove exostoses if the work can be done with a small saw.

DON'T use force in using a saw. Simply guide it and allow it to do the cutting.

DON'T fail to open an abscess of the septum at the earliest opportunity. You may thereby prevent destruction of the cartilage and deformity of the nose.

DON'T remove polypi with the forceps. Use a wire écraseur and cut through the pedicle by turning the screw. DON'T pull.

DON'T straighten a deflected septum by fracturing and replacing until you have prepared the nasal cavity on the concave side for the encroachment on its calibre. The inferior turbinated bone on this side is generally hypertrophied, in which case a portion of its entire length should be removed.

DON'T be in too great haste to plug the nose in cases of hemorrhage after operation. The most copious hemorrhage will usually cease within fifteen minutes.

DON'T plug until you have ascertained where the blood comes from, and then place the plug so as to make proper pressure on the bleeding points.

DON'T plug with anything except iodoform gauze.

DON'T attempt to arrest epistaxis not due to traumatism by astringent injections. Find the bleeding point and touch it with the galvano-cautery.

DON'T forget to examine for adenoids in the pharyngeal vault by introducing the finger through the mouth up behind the soft palate.

DON'T attempt to treat adenoids by astringents or caustics. The Gottstein curette and the Quinlan forceps will remove them thoroughly. The finger of the operator, introduced behind the soft palate into the pharyngeal vault, will not only locate accurately the smallest growth, but will determine when all are removed. Don't leave the smallest particle behind.

DON'T forget that enlarged tonsils obstruct the posterior nares and are a prolific source of nasal and aural disease.

DON'T neglect the tonsils in cases of mouth breathing pointing to nasal obstruction. If they are enlarged remove them with the guillotine or destroy them with the galvano-cautery.

DON'T give a general anæsthetic for operations in the nose. You need a good light, and the patient's assistance in removing blood clots. Besides, the added dangers of an anæsthetic, though slight, should be avoided. Cocaine, properly used, is sufficient for nearly all intranasal operations.

DON'T neglect constitutional treatment in syphilis of the nose. Tertiary syphilis, the form usually met with, requires large doses of the iodide. Locally, the best treatment is iodoform in spray.

DON'T rely exclusively upon topical means in treating affections of the nose. Tonics are always indicated when the general system is at fault.

DON'T expect to cure nasal catarrh in an habitual smoker.

## Figures and Tables

**Figure f1:**